# Quantitative anti-PA IgG ELISA; assessment and comparability with the anthrax toxin neutralization assay in goats

**DOI:** 10.1186/1746-6148-9-265

**Published:** 2013-12-27

**Authors:** Okechukwu C Ndumnego, Jannie Crafford, Wolfgang Beyer, Henriette van Heerden

**Affiliations:** 1 Department of Veterinary Tropical Diseases, University of Pretoria, Onderstepoort 0110, South Africa; 2 Institute of Environmental and Animal Hygiene, University of Hohenheim, Stuttgart, Germany

**Keywords:** Protective antigen, Indirect ELISA, Toxin neutralization assay, Anthrax, Immunoglobulin, Sterne vaccine, Goats

## Abstract

**Background:**

Presently, few data exist on the level and duration of anti-protective antigen (PA) IgG in vaccinated livestock. Various adaptation of enzyme-linked immunosorbent assays (ELISAs) have been developed in studies to assess immune response following vaccination, albeit mostly in laboratory rodent models. The quantitative anti-anthrax IgG ELISA in this study describes a method of enumerating the concentration of anti-PA specific IgG present in sera of immunized goats, with the aid of an affinity-purified caprine polyclonal anti-anthrax PA-83 IgG standard. This was compared with the anthrax toxin neutralization assay (TNA) which measures a functional subset of toxin neutralizing anti-PA IgG.

**Results:**

The measured concentrations obtained in the standard curve correlated with the known concentration at each dilution. Percentage recovery of the standard concentrations ranged from 89 to 98% (lower and upper asymptote respectively). Mean correlation coefficient (r^2^) of the standard curve was 0.998. Evaluation of the intra-assay coefficient of variation showed ranges of 0.23-16.90% and 0.40-12.46% for days 28 and 140 sera samples respectively, following vaccination. The mean inter-assay coefficient of variation for triplicate samples repeated on 5 different days was 18.53 and 12.17% for days 28 and 140 sera samples respectively. Spearman’s rank correlation of log-transformed IgG concentrations and TNA titres showed strong positive correlation (r_s_ = 0.942; p = 0.01).

**Conclusion:**

This study provides evidence that an indirect ELISA can be used for the quantification of anti-anthrax PA IgG in goats with the added advantage of using single dilutions to save time and resources. The use of such related immunoassays can serve as potential adjuncts to potency tests for Sterne and other vaccine types under development in ruminant species. This is the first report on the correlation of polyclonal anti-anthrax PA83 antibody with the TNA in goats.

## Background

*Bacillus anthracis* is a spore-forming bacterium that causes anthrax primarily in herbivorous animals but also affecting other mammalia including humans to a lesser extent [1]. The virulence factors of *B. anthracis* are encoded on the pXO1 and pXO2 plasmids. The pXO1 plasmid carries the genes *pagA*, *lef*, and *cya* that encode the protective antigen (PA), lethal factor (LF), and oedema factor (EF), respectively [2]. The term “protective antigen” was derived because of the protein’s ability to elicit a protective immune response against anthrax [3]. Individually, none of these proteins are toxic, but PA combines with EF to form the oedema toxin (ET). Similarly, PA in combination with LF forms the anthrax lethal toxin (LT) [2,4,5]. The pXO2 plasmid codes for the anti-phagocytic poly-gamma-D-glutamic acid (PGDA) capsule which protects the bacteria against phagocytosis, or consumption by defensive cells of the immune system. Various studies have shown that without its capsule, the bacteria can be phagocytized and destroyed [6,7]. Attenuated strains that lack either of the plasmids have a reduced virulence [1].

The current anthrax veterinary vaccine is the attenuated *B. anthracis* 34 F2 strain which was developed in 1937 by Max Sterne at Onderstepoort in South Africa [8]. Sterne derived a rough variant of virulent *B. anthracis* by culturing the organism on serum agar in elevated CO_2_ atmosphere. The attenuation of this strain was subsequently shown to be due to loss of the capsule-encoding pXO2 plasmid [9]. Compared to wild type *B. anthracis* strains, the Sterne strain is relatively avirulent but immunization of animals with the strain is able to stimulate a protective immune response. The Sterne vaccine consist of 1–5 × 10^6^ spores per dose suspended in glycerine and is administered subcutaneously [10]. Ivins *et al*. [11] in his study concluded that the nontoxigenic Pasteur vaccine lacking the pXO1 plasmid did not provide protection and that attenuated, live *B. anthracis* strains must produce the toxin components to enable successful immunization. Presently, the Sterne live spore vaccine is the most widely used strain for immunization of animals against anthrax.

The efficacy of the Sterne vaccine was originally assessed by virulent challenge of vaccinated sheep, guinea pigs, cattle, horses, goats and rabbits. These trials were not comparable as the vaccine/challenge doses and strains varied in the different animal species [1]. Furthermore, adverse reactions in goats vaccinated with the Sterne vaccine was reported by Sterne [12]. Lincoln *et al*. [13] indicated that the susceptibility of animal species to anthrax is proportional to their susceptibility to the anthrax toxin. This focused research on the development and improvement of serological tests to assess protection provided by anthrax vaccines. Serological tests before the 1980s lacked sensitivity and/or specificity [14]. This problem was surmounted by the purification of the PA component of the anthrax toxin [15,16] and the application of the enzyme-linked immunosorbent assay (ELISA) that was highly sensitive in detecting anti-anthrax antibodies. ELISAs have been widely used in the diagnosis of anthrax and the development of new anthrax vaccines [17,18]. However, little data exists on the prevalence, the level of- and the duration of anti-anthrax antibodies in vaccinated livestock. Various versions and adaptations of ELISAs have been developed in several studies to assess the immune response following vaccination, though mostly in laboratory rodent models. Evaluating immune response in vaccinates by a titre-based ELISA method has been used previously in vaccine potency and immunogenicity studies in the veterinary field [19-21]. The quantitative anti-anthrax IgG ELISA in this study describes a method of enumerating the concentration of anti-PA specific IgG present in sera of immunized goats, with the aid of an affinity-purified caprine polyclonal anti-anthrax PA83 IgG standard. This method will be compared with the anthrax toxin neutralization assay (TNA).

The TNA is a technique developed to measure the ability of antibodies in sera of immunized animals to neutralize the PA and its contribution to LT cytotoxicity for certain sensitive cell lines [22-24]. This technique is species independent and has been standardized for use with multiple species [25-27]. Therefore this study involved the adaptation and optimization of the quantitative anti-anthrax IgG ELISA in goats and its comparison with the standard anthrax TNA. It attempts to address the feasibility of the use of a quantitative anti-PA antibody ELISA in evaluating the immunoglobulin kinetics in an immunized caprine model.

## Results

### Evaluation of characteristics of reference standard 4-parameter curve

**Figure 1:**
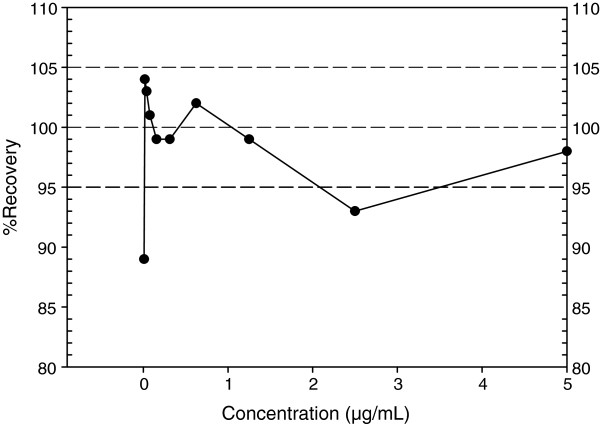
**Percentage recovery (%Re) plots for known concentrations of the reference anti-PA IgG standard.** %Re indicates percentage recovery for known IgG concentrations of the standard that were tested in the ELISA using a 4-parametre logistic curve model. [%Re = 100(BC/NC), where BC and NC represent the back-calculated and nominal (known) concentrations respectively].

**Figure 2:**
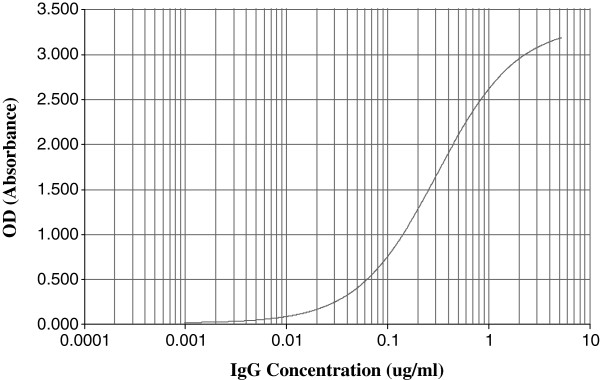
**Anti-PA IgG 4-parameter logistic curve model derived for a quantitative indirect ELISA using the Gen 5 software.** Data were derived from a reference standard consisting of affinity-purified caprine polyclonal anti-anthrax PA83 IgG fraction with dilutions from 5–0.0098 μg/ml.

Data from 22 reference standard curves made up of 10, two-fold serial dilutions from 5–0.0098 μg/ml were calculated to be A = 0.0270 ± 0.0132; B = 1.151 ± 0.0467; C = 0.4268 ± 0.1576 and D = 3.473 ± 0.1154 (±SD). A good %Re was recorded for the nominal concentrations (Figure 1) with the only exception being at the lower asymptote (89%) for the 0.0098 μg/ml concentration though well within the established acceptable range of 80% for lower limit of quantitation [28]. The mean correlation coefficient (R^2^) of the standard curves was 0.9998 (Figure 2) with ranges between 0.9998 and 1.0000.

### Precision of assay

Evaluation of the intra-assay coefficient of variation showed ranges from 0.23 - 16.9% and 0.40 - 12.46% for day 28 and 140 sera respectively. Average inter-assay CV was 18.53% for day 28 sera and 12.17% for day 140 sera collection (Table 1). Slightly higher CV values were observed with the day 28 sera compared to sera collected 140 days after vaccination.

**Table 1:** Mean IgG concentrations for caprine sera collected on days 0, 28 and 140 days respectively

	**Day 0**	**Day 28**	**Day 140**
**Animal**	**IgG μg/mL (CV)**	**IgG μg/mL (CV)**	**IgG μg/mL (CV)**
#8172	3.2534 (52)	221.8 (16.8)	56.1 (14.9)
#8173	1.1405 (11.5)	719.6 (16.2)	166.2 (15.1)
#8174	0	546.8 (16.3)	102.7 (11.3)
#8175	2.3788 (65.5)	585.9 (17.1)	59.8 (9.9)
#8176	0.8115 (83.9)	584.2 (19.0)	106.3 (11.4)
#8178	1.7455 (14)	464.4 (18.1)	24.7 (13.1)
#8179	1.5884 (64.8)	268.3 (20.1)	55.9 (12.4)
#8180	0	227.2 (16.6)	28.0 (14.5)
#8181	0.115	482.8 (22.3)	41.8 (10.7)
#8182	0	719.0 (22.8)	92.4 (8.44)

Five assays were done in triplicates for each serum sample.

### Correlation between quantitative ELISA and toxin neutralization assay

**Figure 3:**
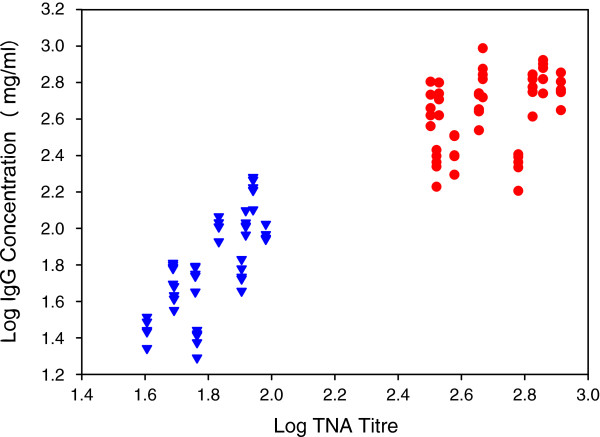
**Scatter plots of log TNA titres by log ELISA μg/ml from sera of ten goats analysed over five different days with the quantitative indirect ELISA (in triplicates) and once with the TNA (duplicates) on 28 (circles) and 140 (triangles) days after vaccination with the Sterne vaccine.** Spearman’s rank correlation of the log-transformed IgG concentrations and TNA titres showed a positive correlation, (r_s_ = 0.942; p = 0.01).

We investigated the ability of the collected caprine sera to protect susceptible mouse macrophages from the deleterious effects of anthrax lethal toxin using the TNA. The toxin neutralizing titres of sera from individual animals at specific time-points were compared with the corresponding IgG concentration using the Spearman’s rank correlation test. The scatter plot (Figure 3) indicates a similar correlation between the log TNA and IgG values at days 28 and 140 post-vaccination respectively. Spearman’s rank correlation of the log-transformed IgG concentrations and TNA titres showed strong positive correlation (r_s_ = 0.942; *p* = 0.01). TNA values for the day 0 sera were negligible, being below the starting dilution of 50 for the assay (not shown).

## Discussion

Very little data is available on the kinetics of anthrax antibodies in vaccinated livestock and the duration of immunity. The use of immunoassays could serve as adjuncts to potency tests for the Sterne and other vaccine types under development. In this study we have modified and adapted an ELISA [29] for the quantitation of anti-PA immunoglobulins in goats vaccinated with the Sterne live spore vaccine. This study investigated the feasibility and characteristics of an IgG-quantitative indirect-ELISA using a standard curve. This approach has been used in the field of vaccine development albeit with different model species [30,31]. Correlation between ELISA-measured IgG levels and TNA derived neutralization titres have been shown previously in various laboratory species [24,32] but not in a ruminant species. Therefore the ELISA was also compared to the TNA.

PA is the most essential component of live, inactivated or protein-based anthrax vaccines [33] and strains of *B. anthracis* without the toxin producing plasmid, pXO1, have failed to confer protective immunity to animals with exception to mice [33,34]. More so, numerous studies have shown PA to be able to induce a protective response in experimental models of infection [35]. Thus, evaluation of anti-PA antibodies in sera of immunized animals following vaccination is an important step in the evaluation of vaccine efficacy due to the essential role played by PA in anthrax pathogenesis. Anti-PA antibodies have also been shown to suppress germination of spores and to enhance phagocytosis of spores by macrophages and thus preventing the establishment of infection [36,37]. Seroconversion to PA specific antibodies following vaccination could be an indication of the immune status in vaccinated herbivores, as already indicated in laboratory animals [29,36].

Our study evaluated the standard curve on each ELISA plate and found a high repeatability with an average R^2^ of 0.9998 (Figure 2). A sound standard calibration curve is essential in the development and assessment of quantitative assay characteristics such as accuracy and precision [38]. The key factor is the level of agreement of known standard calibrator concentrations with back-fitted concentrations with the latter read of the fitted standard curve as if they were unknown samples [39,40]. The drifting of the %Re from the ideal 100% can be attributed to stronger effects of nonspecific binding at the lower asymptote as antibody concentration reduces dramatically. The same effect is seen at the upper asymptote possibly due to the near infinite antibody concentration (maximum response) though not as pronounced as seen in the lower asymptote. It can also be posited that the upper and lower limits of detection of antibodies of this assay are very close to the respective asymptotes of the standard curve (5 μg/ml and 0.0098 μg/ml respectively). Although %Re of the standard at both asymptotes is not optimal, this effect can be minimized by the limiting of the calibration to concentrations within the ideal 100% recovery level. Crucially, the assessment of the suitability of a standard curve for any immunoassay should be done early in the assay development, as a sound calibration curve is central to the development of sound assay characteristics [38]. This is also very important in the further development of the assay since the quantification of antibodies in test sera is derived from the standard curve [31,41]. Moreover, the inclusion of a serially diluted standard reference on every ELISA plate serves as a normalization and/or internal control for the individual assays.

The OD_405_ values of the assay blank wells also fell within the acceptable level for an early stage ELISA (averaging 0.069). The use of skimmed milk powder instead of the conventional foetal calf serum as previously described [42] improved the specificity of the ELISA (low background noise). Non-specific binding or low background noise in an ELISA system has been attributed to the use of sera as a blocking agent in ELISA systems [42]. This phenomenon was reduced with the use of skimmed milk powder as the blocking agent of choice.

With the IgG quantitiative ELISA in goats more sera samples can be assessed on each ELISA plate when compared to the conventional titre-based ELISA where end-point titrations for each serum sample are performed in rows on the plates. Good reproducibility of the data was obtained over five different assays with the CV within acceptable limits. The CV between different runs (performed on different days) was much lower for sera collected on day 140 compared to samples collected on day 28. This could be due to the presence of more robust antibodies with higher antigenic affinities.

Assessment of LT neutralizing antibodies by the TNA in various species has been shown to correlate with survival in various laboratory animal species [43-45]. These studies evaluated anthrax PA-based vaccines in laboratory rodents like guinea pigs, rabbits and mice and found correlation between toxin neutralizing antibodies and protection [43-45]. This study is the first to quantify anti-PA immunoglobins in goats using the live Sterne spore vaccine in a host animal species. It is not known if a correlation between toxin neutralizing antibodies and protection will be observed with a virulent *B. anthracis* challenge of the vaccinated goats. Crucially, we have observed a significant positive correlation between ELISA-derived IgG concentrations and TNA titres in goats. This is an important step in further studies exploring the correlates of protection against virulent spore challenge in goats and evaluation of test vaccines. More so, it will elucidate more on the possible role of anti-PA antibodies in goats, as little is known in this regard.

## Conclusion

In summary, though not exhaustive in its approach, this study indicates that a reliable IgG quantitative ELISA can be useful for vaccine studies in goats. The assay has the advantage of a reference standard in every plate which gives a measure of good internal control, in addition to the positive and negative controls. The feasibility of a full, long term validation of the assay seems favourable and should serve as the prelude to its use in anthrax vaccine research and production in goats.

## Methods

### Recombinant PA (rPA)

Purified recombinant antigen (rPA83) [29] with concentration of 1450 μg/ml in bicarbonate buffer (0.05 M, pH 9.5) and stored at −20°C was obtained from Dr. Wolfgang Beyer, Institute of Environmental and Animal Hygiene, University of Hohenheim, Stuttgart.

### Immunization

Ten naïve, age-matched Boer goats were housed at the experimental animal facility of Onderstepoort Biological Products (OBP), South Africa and were allowed an acclimatization period of 14 days. The goats were vaccinated subcutaneously on day 0 with 1 ml of the commercial *Bacillus anthracis* Sterne 34 F2 vaccine (1 × 10^6^ spores/ml) according to the manufacturers’ instruction (OBP) and monitored throughout the duration of the trial. Blood was collected on days 0, 28 and 140 respectively. The harvested sera were aliquoted into 1 ml cryovials and stored at −20°C till use. Serum from a goat revaccinated one year after the first immunization served as the positive control in the ELISA. The animal research was approved by the animal ethics committee (AEC) of the University of Pretoria, South Africa (protocol approval number V41-10) and the Department of Agriculture, Forestry and Fisheries, South Africa under the animals disease act (act 36 Section 20, 1984).

### Quantitative indirect anti-PA IgG ELISA

An antibody reference standard that consisted of affinity-purified caprine polyclonal anti-anthrax PA83 IgG fraction (10 mg/ml) was obtained from Innate Therapeutics (Auckland) and stored at −20°C until used. This indirect ELISA measured anti-PA83 specific IgG in 96-well microtitre plates (Maxisorp Nunc-immuno plate, Germany) coated with rPA83 as the capture antigen. Each plate contained one duplicate negative control (from unvaccinated goat), three duplicates positive control at high, medium and low concentrations, four blanks and 20 test sera in triplicates at a 1:400 dilution. The reference standard was titrated in duplicate in a 2-fold dilution series.

Individual wells of the plates were coated with 100 μl of rPA diluted to 5 μg/ml in bicarbonate buffer [46]. After 24 hours incubation at 4°C, the plates were washed twice with PBS containing 0.05% Tween 20 (Merck, Pretoria, South Africa) (PBST) using a Biorad PW 40 washer (Marnes-La-Coquette, France). Plates were blocked with 200 μl of PBST containing 10% skimmed milk powder (Oxoid, Hampshire, England) (PBSTM) and incubated for 1 hour at room temperature. The plates were washed as before and 100 μl of the test sera as well as the reference standard (5–0.0098 μg/ml) were diluted in PBSTM and added to the respective wells. Blank wells received only 100 μl of PBSTM. The plates were then incubated for 30 minutes at room temperature on a rotatory plate shaker (200 rpm) (Titretek® Flow Labs, Irvine, UK). After five washes, 100 μl of horseradish peroxidase-conjugated rabbit anti-goat IgG (Invitrogen, Camarillo, CA, USA) diluted to 1:4000 in PBSTM was added to every well and incubated for 30 minutes as before. After the incubation, the plates were washed five times before adding 100 μl of the enzyme substrate 2,2-Azino-bis(3-ethylbenzthiazoline-6-sulfonic acid) diammonium salt (Sigma, Steinheim, Germany) to each well. Plates were incubated for 40 minutes in the dark. Absorbance was measured at 405 nm using a Biotek power wave XS 2 reader (Winooski, USA). Plates were blanked with the wells containing PBSTM only. The anti-PA83 IgG concentration of the sera were calculated from the corresponding reference standard curve on the respective ELISA plates using the 4-parameter logistic regression equation in the Gen5 data analysis software (Biotek Instruments, USA).

### Anthrax toxin neutralization assay (TNA)

The anthrax TNA was performed using the mouse macrophage cell line J774A.1 (European collection of cell cultures, ECACC) as previously described [25] with slight modifications. 96-well flat-bottomed tissue culture plates (Greiner Bio One, Germany) containing 8 × 10^4^ macrophages/well in DMEM (Life Technologies, USA) and 10% FCS (Life Technologies, USA) were incubated overnight at 37°C and 5% CO_2_. Goat sera were doubly diluted (1:50 to 1:6400) in culture medium containing PA and LF (List Biological Laboratories Inc., Campbell, CA) at concentration of 500 ng/ml and 400 ng/ml (lethal toxin, LT) respectively. The sera/LT mixture was incubated for one hour at 37°C and 5% CO_2_ before adding to overnight cultured cells (after discarding medium) and incubated for three hours. Each serum sample was tested in duplicate. Following incubation, 25 μl of 5 mg/ml MTT (3-(4,5 dimethylthiazol-2-yl)-2,5-diphenyltetrazolium bromide; Life Technologies, USA) was added to each well and incubated in the dark at 37°C and 5% CO_2_. After two hours incubation, the cells were lysed with prewarmed (37°C) acidified isopropanol (90% isopropanol, 0.5% SDS w/v, 25 mM HCl, pH 4.7) by vigorously pipetting up and down to solubilize the formazan dye. The plates were rested for five minutes and the absorbance readings taken at 540 nm with a Biotek power wave XS2 reader. Each assay included a single dilution series of positive control serum from a goat hyper immunized with the Sterne live spore vaccine. Three wells in each assay receiving LT served as blanks, another triplicate of wells (with cells) received only LT as toxin control while only culture media was placed in two wells (medium control). The neutralization titre of each test serum was calculated by: NT_50_ = (sample – toxin control)/(medium control – toxin control) × 100 and expressed as the reciprocal of the highest serum dilution neutralizing 50% of the LT cytotoxicity. NT_50_ data were obtained using the Gen5 data analysis software (Biotek Instruments, USA).

### Statistical analysis

For determination of assay precision, serum samples were tested in triplicates on five different days. Mean absorbance values, standard deviation and coefficient of variation (CV) for each duplicate dilution of test samples, controls and r^2^ of standard curve were calculated using the Gen5 software. The Gen5 program 4-parameter logistic standard curve is delineated by the formula; Y = (A-D)/(1+ (X/C)^B^) + D where ‘Y’ is the optical density (OD) of the test/control sample, ‘A’ is the response at zero concentration, ‘B’ is the measure of the slope curve at its inflection point, ‘C’ is the value of X at inflection point and ‘D’ is the response at infinite concentration. This equation defines the relationship between obtained absorbance values and the known concentrations of a reference standard [30,47].

In evaluating the assay standard curve, we expressed the predicted standard concentrations as a percentage recovery (%Re) at each concentration level, %Re = 100(BC/NC), where BC and NC represent the back-calculated and nominal (known) concentrations respectively. Curve fitting was done using SigmaPlot (Systat software Inc, San Jose, USA) and data were analysed with the statistical software package SPSS Version 21 (IBM SPSS Statistics; IBM Corporation, Armonk, New York, USA). Correlations at the *p* ≤ 0.01 level was considered statistically significant.

## Competing interests

The authors declare that they have no competing interests.

## Authors’ contributions

Conceived and designed the experiments: WB, HvH, OCN. Performed the experiments: OCN. Analysed and interpreted the data: OCN, JC, WB, HvH. Drafted the manuscript: OCN. All authors read, edited and approved the final manuscript.
